# The Role of Vitamin D and Vitamin D Binding Protein in Chronic Liver Diseases

**DOI:** 10.3390/ijms231810705

**Published:** 2022-09-14

**Authors:** Tudor Lucian Pop, Claudia Sîrbe, Gabriel Benţa, Alexandra Mititelu, Alina Grama

**Affiliations:** 1 2nd Pediatric Discipline, Department of Mother and Child, “Iuliu Hatieganu” University of Medicine and Pharmacy, 400012 Cluj-Napoca, Romania; 2 2nd Pediatric Clinic, Emergency Clinical Hospital for Children, 400177 Cluj-Napoca, Romania

**Keywords:** vitamin D, vitamin D binding protein, chronic liver diseases, fibrosis, non-alcoholic fatty liver disease, children

## Abstract

Vitamin D (calciferol) is a fat-soluble vitamin that has a significant role in phospho-calcium metabolism, maintaining normal calcium levels and bone health development. The most important compounds of vitamin D are cholecalciferol (vitamin D3, or VD3) and ergocalciferol (vitamin D2, or VD2). Besides its major role in maintaining an adequate level of calcium and phosphate concentrations, vitamin D is involved in cell growth and differentiation and immune function. Recently, the association between vitamin D deficiency and the progression of fibrosis in chronic liver disease (CLD) was confirmed, given the hepatic activation process and high prevalence of vitamin D deficiency in these diseases. There are reports of vitamin D deficiency in CLD regardless of the etiology (chronic viral hepatitis, alcoholic cirrhosis, non-alcoholic fatty liver disease, primary biliary cirrhosis, or autoimmune hepatitis). Vitamin D binding protein (VDBP) is synthesized by the liver and has the role of binding and transporting vitamin D and its metabolites to the target organs. VDBP also plays an important role in inflammatory response secondary to tissue damage, being involved in the degradation of actin. As intense research during the last decades revealed the possible role of vitamin D in liver diseases, a deeper understanding of the vitamin D, vitamin D receptors (VDRs), and VDBP involvement in liver inflammation and fibrogenesis could represent the basis for the development of new strategies for diagnosis, prognosis, and treatment of liver diseases. This narrative review presents an overview of the evidence of the role of vitamin D and VDBP in CLD, both at the experimental and clinical levels.

## 1. Introduction

In recent years, vitamin D or calciferol has become a subject of intense scientific investigation and has found a new place under the sun. Vitamin D is a fat-soluble vitamin that has a major role in phospho-calcium metabolism, maintaining normal calcium levels and bone health development [1,2]. Vitamin D has two essential compounds: vitamin D3, or cholecalciferol and vitamin D2 or ergocalciferol. Both forms of vitamin D play significant roles in the body, protecting it against rickets or bone demineralization, hypertension, cancers, or autoimmune disorders [1,2,3,4]. It also plays a crucial role in anti-infective defense through the anti-inflammatory, immunomodulatory, proapoptotic, and antiangiogenic effects. In the body, vitamin D is transported by a molecule, Gc-globulin or vitamin D binding protein, which also plays an essential role in maintaining the body’s homeostasis through its anti-inflammatory role. This narrative review presents an overview of the evidence of the role of vitamin D, its metabolites, and vitamin D binding protein (VDBP) in chronic liver diseases (CLD), both at the experimental and clinical levels [1,2,3,4]. For this purpose, we reviewed the existing literature data about the involvement of vitamin D and VDBP in chronic hepatitis, hepatic steatosis, fibrosis, cirrhosis, or liver transplantation.

## 2. Vitamin D Metabolism and Its Action

Vitamin D, through its metabolites, increases serum calcium concentrations through stimulation of active intestinal calcium absorption, mobilizes calcium from the bones when it is absent from the diet, and stimulates osteoblasts to produce receptor activator nuclear factor-κB ligand (RANKL), which activates resting osteoclasts for bone resorption (osteoclastogenesis) [1]. Vitamin D and parathyroid hormone permanently control calcium and phosphorus homeostasis [2,3,4]. Most of the calcium is absorbed in the distal intestine (70–80% of the ingested calcium) but also in the colon, two organs rich in vitamin D receptors (VDR), calcium transport protein 1 (CaT1), calbindin-D9K, and transient receptor potential vanilloid type 6 (TRPV6). These membrane proteins transport calcium through the cell [2,3,4]. In the kidney, reabsorption processes occur in the distal renal tubules, where about 1% of the calcium is reabsorbed. It seems small but considering that the total amount of calcium filtered in a day reaches 7 g, it is an essential process in the phospho-calcium balance [1].

**Figure 1:**
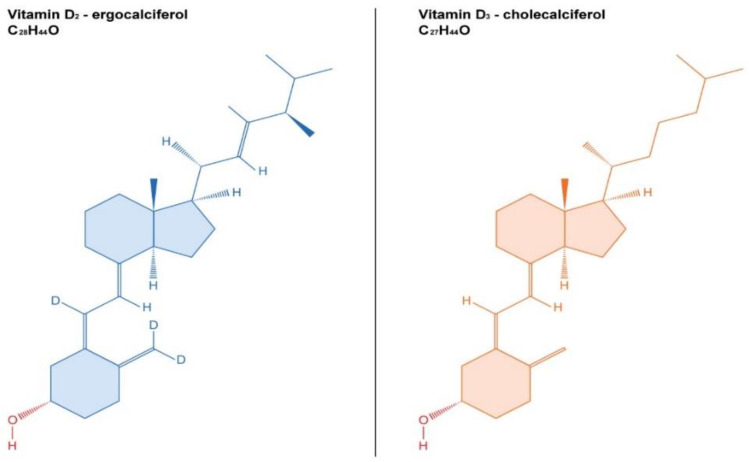
Chemical structure of the vitamin D2, ergocalciferol (C_28_H_44_O), and vitamin D3, cholecalciferol (C_27_H_44_O).

**Figure 2:**
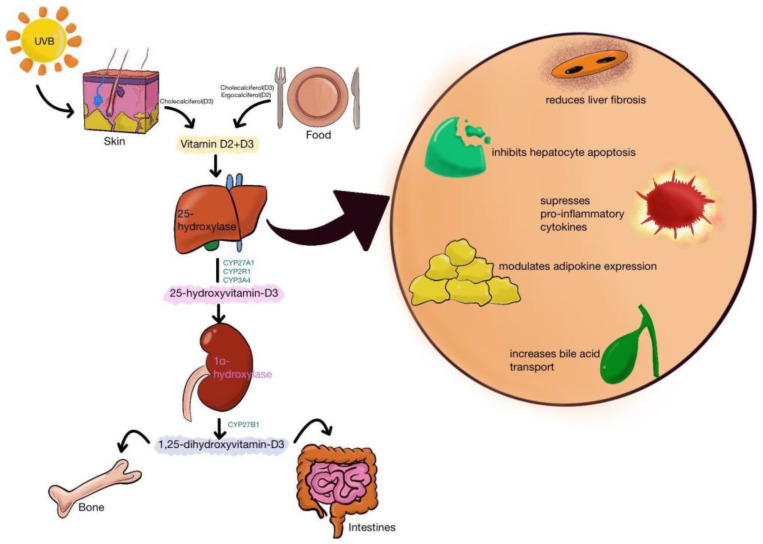
Vitamin D2 and Vitamin D3 metabolism and its different roles in the liver: reduce fibrosis, inhibit hepatocytes apoptosis, suppress pro-inflammatory cytokines, modulate adipokines expressions, and increase bile acid transport.

The most important compounds of vitamin D are vitamin D3 (also known as cholecalciferol, VD3) and vitamin D2 (ergocalciferol, VD2) with a similar chemical structure (Figure 1). VD3 is produced in the skin from 7-dehydrocholesterol in the presence of solar UV radiation. It is also found in some foods such as fish, beef liver, eggs, and cheese, milk. In the liver, VD3 is transformed to calcifediol (25-hydroxyvitamin D, 25(OH)D) by CYP2R1 or CYP27A1 (Figure 2). The most important role of VD3 is to increase calcium uptake by the intestine. This process occurs through two mechanisms, one energy-dependent and a passive paracellular pathway [2,3,4,5]. 25(OH)D is converted to calcitriol (1,25-dihydroxyvitamin D or 1,25(OH)_2_D3) through the action of 1-hydroxylase (CYP27B1). All these metabolites will bind vitamin D binding protein (VDPB) and less to albumin and will be transported to the tissues. Here, the action of calcitriol is mediated by the VDR, a nuclear receptor in every cell of the body. Both 25(OH)D and calcitriol are catabolized by CYP24A1, a member of the cytochrome P450 [6]. However, calcitriol has a very high affinity for VDR, acting through a series of cell-signaling reactions or as a ligand-activated transcription factor resulting in immediate responses in the target cells [7].

VD2 comes only from external sources (the plant sterol ergosterol) and is not produced in the human body. When compared to VD3, it has lower effects but is not negligible. It is used as a medication in many countries because it prevents and treats vitamin D deficiency [8,9]. Like VD3, it is also an inactive product that requires two hydroxylations to become active. The first reaction is in the liver by CYP2R1 to form 25-hydroxyergocalciferol (25(OH)D2), and the second one in the kidney by CYP27B1 to form the active 1,25-dihydroxyergocalciferol (ercalcitriol or 1,25(OH)_2_D2). These new metabolites will activate VDR, and they will exercise their action. VD2 supplementation seems to be extremely important in patients with end-stage renal disease, improving bone and mineral metabolism [10]. Generally, in the first stage, exogenous calcium is used and only in its absence is endogenous calcium used [6].

Vitamin D2 and D3 have many essential functions. A sufficient level of 25(OH)D in the body will protect against failed bone mineralization and rickets among children and osteomalacia among adults. It also decreases the risk of osteoporosis, hypertension, cancer, or autoimmune diseases in adults. Vitamin D, through its metabolites, stimulates the immune response in children and adults, having a significant role in anti-infective defense. Many studies report the importance of vitamin D in inhibiting the carcinogenesis process and against progression to metastatic disease, protecting against breast, colon, skin, stomach, or prostate cancers. Vitamin D and its metabolites also have important anti-inflammatory, immunomodulatory, proapoptotic, and antiangiogenic effects [1,11,12,13].

Vitamin D3 and D2 are stored in adipose tissue where numerous VDRs are also expressed, being involved in the regulation process of metabolic disorders. Thus, vitamin D is an essential catalyst for energy homeostasis and glucose metabolism, influencing insulin secretion, glucose levels, or inflammation. Thus, vitamin 25(OH)D deficiency is one of the predisposing factors for fatty metabolic diseases, such as obesity, diabetes mellitus (DM), atherosclerosis, non-alcoholic fatty liver disease (NAFLD), or multiple sclerosis [14].

Vitamin D3 and D2 interfere with many detoxification processes in the liver by stimulating P450 cytochromes expression (i.e., CYP3A4, CYP2B6, and CYP2C9) and promoting normal liver recovery after partial hepatectomy by increasing intracellular calcium flow, control DNA polymerase α activity, and nuclear protein kinase activity. VDRs are localized in the cell nucleus and are found in most body tissues, like the liver, kidney, thyroid, adrenal glands, gastrointestinal tract, breast, or skin. One of the most important roles of VDR is to inhibit CYP7A1 mRNA expression and bile acid synthesis, thereby protecting hepatocytes against cholestasis [15]. Vitamin D has important antiproliferative and antifibrotic effects on liver fibrosis by inhibiting the expression of proliferation and profibrotic markers in hepatic stellate cells and excessive deposition of extracellular matrix components [16].

However, in addition to vitamin D, its transport molecule, named Gc-globulin, plays a significant role in maintaining this homeostasis. Gc-globulin is a serum α2-globulin with a molecular weight of 52–59 kDa. Gc-globulin was first described by Hirschfeld in 1959. Over time, the name of this protein has changed with the discovery of its many roles, so it was called vitamin D binding protein (VDBP) due to its role in vitamin D transport, or macrophage-activating factor (Gc-MAF/DBP-MAF) after discovering its activity of stimulating macrophages [17]. VDBP is a protein encoded by the DBP gene, located on the long arm of chromosome 4 (4q12-q13) and expressed in various tissues. The gene encodes the family of multifunctional plasma proteins belonging to the albumin superfamily of binding proteins, which also includes albumin, α-fetoprotein (AFP), and α-albumin/afamin (AFM) [18,19]. There are more than 120 variants of VDBP, the most common phenotypes being DBP1F, DBP1S, and DBP2, linked to rs7041 and rs4588 polymorphisms [20]. Homozygotes for rs7041 and rs4588 have a high serum concentration of 25(OH)D after supplementation with vitamin D [17,21].

**Figure 3:**
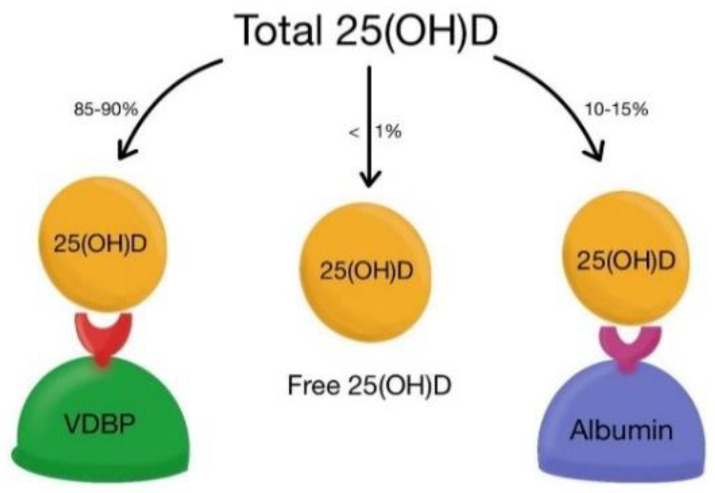
Total 25(OH)D in serum is bound to vitamin D binding protein (VDBP), 85–90%; albumin, 10–15%; and <1% circulates free. Bioavailable vitamin D refers to vitamin D3 and D2, which are not bound to VDBP (Adapted from Fernando M, [31]).

**Figure 4:**
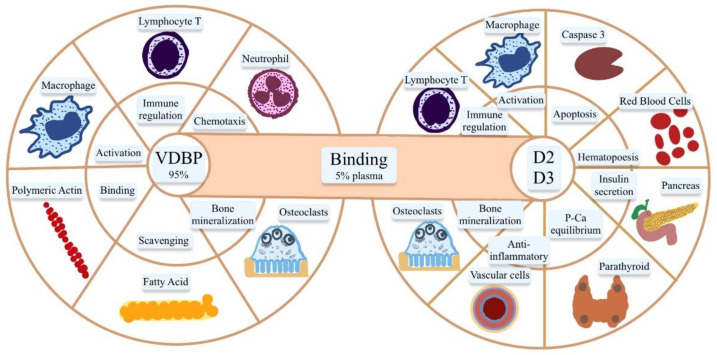
The roles of vitamin D2 and D3 and VDPB. Only 5% of VDBP is found in plasma, bound by 25(OH)D, the main active metabolite of VD2 and VD3 subunits. The rest of the 95% is found in different tissues and organs where different functions are performed. An important anti-inflammatory roll, through the different effects on lymphocytes, macrophages, or neutrophils, regulates lipid metabolism by binding fatty acids and significantly reducing the risk for atherosclerosis or insulin resistance, prevents disseminated intravascular coagulation by stimulating actin degradation, or regulates bone resorption by stimulating osteoclast activity. Vitamin D2 and D3m through their metabolites, perform a lot of functions in bone mineralization; autoimmune disorders or different cancers; anti-infective defense by its anti-inflammatory role; and protection against obesity, diabetes mellitus, atherosclerosis, non-alcoholic fatty liver disease, liver fibrosis, or multiple sclerosis [14,15,16,17,18,19,20,21,22,23,24].

VDBP is synthesized by the liver and has the role of binding and transporting vitamin D and its metabolites to the target organs (Figure 3 and Figure 4) [4]. The affinity of VDBP is the highest for 25(OH)D and less for vitamin D itself or 1,25(OH)_2_D. Also, the affinity is greater for VD3 metabolites than for those of VD2. Only 5% of total plasma VDBP is bound to vitamin D, the remaining 95% being found in different organs (heart, brain, lungs, kidneys, spleen, tests, and uterus), where various functions are performed such as increased neutrophil chemotaxis, T cell response, activity as VDBP-macrophage activating factor (DBP-MAF), binding fatty acids, vitamin D metabolites, or actin scavenging [18,19,20,22,23,24]. Other functions of VDBP are the stimulation of the activity of osteoclasts and bone resorption [22,23,24]. Therefore, VDBP plays an important role in inflammatory response secondary to tissue damage, being involved in the degradation of actin. Actin is a protein that forms part of the cytoskeleton, which is involved in cell motility and maintaining the cell’s shape. After an injury that causes cell necrosis or tissue damage, actin is released into the circulation in large quantities, forming long filaments (F-actin). These, together with the coagulation factor V, trigger disseminated intravascular coagulation. Physiologically, the mechanism of action of VDBP is actin binding and scavenging [24,25,26,27]. In the absence of VDBP, actin remains free, favoring platelet aggregation and thrombus formation. VDBP binds to circulating neutrophils and stimulates their chemotactic activity, intensifies C5a-mediated signals, increases macrophage activity at the site of tissue injury, and amplifies macrophage apoptosis processes by inducing caspase [20,22,23,24,25,26,27,28]. It plays an essential role in the uptake and transport of endotoxins in sepsis, suggesting its role in predicting the evolution of the disease in patients with peritonitis. The most important role of VDBP is related mainly to vitamin D3, increasing its biological half-life, thus extending its duration of action, protecting the tissues from its excessive action, limiting its action at the tissue level, and promoting renal reabsorptions [29,30]. The level of VDBP is not correlated directly with vitamin D3 levels. A low level of VDBP is associated with a high bioavailability of D3 in the tissues [26,27,28,29,30].

VDBP is found in smaller quantities in most body fluids (serum, saliva, urine, breast milk, cerebrospinal fluid, seminal liquid, or ascites liquid). The serum concentration of VDBP is between 350 and 500 µg/L, varying during the day (lower in the morning and increase later in the day) [20]. The level of DBP is not influenced by serum vitamin D level, even if it is deficient, correlating only with the 24,25-dihydroxycholecalciferol level, an inactive metabolite of 25(OH)D3. VDBP has a high turnover rate. Research studies on immunodeficiency techniques in adults or animals describe a significant decrease in serum VDBP concentrations in severe tissue damage, considered a prognostic marker in the evolution [18,28].

## 3. Vitamin D in Liver Diseases

Vitamin D is important in cell growth and differentiation, immune function, and cardiovascular and calcium and phosphate homeostasis [32]. Recently, the association between 25(OH)D deficiency and the progression of fibrosis in CLD was confirmed [33], given the hepatic activation process and high prevalence of vitamin D deficiency in these diseases. The causal relationship is uncertain: whether vitamin D deficiency increases the risk for liver fibrosis or CLD is associated with vitamin D deficiency [34,35,36].

The prevalence of vitamin D deficiency (serum level of 25(OH)D under 20 ng/mL) was reported in CLD to range from 64% to 92%. Previously, this deficiency was commonly described in cholestatic disorders due to impaired intestinal absorption. There are reports of vitamin D deficiency in CLD regardless of the etiology (chronic viral hepatitis; alcoholic cirrhosis; NAFLD; primary biliary cirrhosis; PBC; or autoimmune hepatitis, AIH) [37].

Potential mechanisms of VD3 or VD2 deficiency in CLD include reduced exogenous vitamin D sources (diet, limited exposure to sunlight), the intestinal malabsorption of vitamin D due to cholestasis, the reduced production of VDBP and albumin due to liver injury, impaired hepatic hydroxylation, and increased catabolism of 25(OH)D [37]. Besides these causes, the genetic studies identified genetic determinants of vitamin D status: variants at three loci in genes involved in the synthesis, hydroxylation, and transport of VD (7-dehydrocholesterol reductase, DHCR7, CYP27R1 and DBP) [38,39].

**Vitamin D in biliary atresia and fibrosis**. Biliary atresia (BA) is an important cause of cholestasis in infants. Most patients had progressive liver fibrosis despite the hepatic portoenterostomy (HPE) and evolved toward cirrhosis and end-stage liver disease with the need for liver transplantation (LT) [40]. Progressive liver fibrosis is the most important predictor of outcome after HPE in BA [41]. The mechanism of liver fibrosis and cirrhosis in BA includes immune dysregulation, viral infection (cytomegalovirus, CMV), or excessive inflammatory factors [42,43,44,45,46]. Activation of hepatic stellate cells (HSC) through the TGF-signaling pathway and excessive extracellular matrix deposition are essential steps in liver fibrosis [47].

Vitamin D deficiency is common in almost all infants with BA before HPE, reported in 96.3–98.9% of patients [33,41,48], but even after HPE and oral supplementation with vitamin D products [49]. A serum level of 25(OH)D was higher in the jaundice-free post-HPE patients than in those with jaundice after HPE [49,50]. Vitamin D absorption is impaired in cholestatic disorders, but vitamin D activation is also significantly reduced in infants with BA [33].

The role of vitamin D in fibrosis may be explained through its actions as a regulatory factor: Reducing the proliferation and migration of HSC and by binding with VDR with upregulation of CYP2R1 can regulate the activity of the TGF-beta/SMAD signaling pathway in HSCs; inhibits the expression of profibrotic genes such as Col-1alfa1, alfa-SMA, TIMP-1, and deposition of types I and III collagen; and promotes expression of antifibrosis genes such as MMP-2 or MMP9 [16,33,51,52,53,54,55]. 

VDR is not expressed in liver tissue but in nonparenchymal liver cells (HSCs). It was demonstrated that vitamin D deficiency is associated with severe cirrhosis [56], and VDR gene polymorphism increases the risk of cirrhosis in PBC and NAFLD patients [57,58]. It was demonstrated that VDR gene knockout mice could lead to primary liver fibrosis [54].

Vitamin D administration reduces extracellular matrix deposition and attenuates fibrosis in animal models of chronic hepatic injury [16,54,59], but the ability to remediate already-established fibrosis is less promising. 

Vitamin D deficiency in BA patients promotes liver fibrosis [33]. CYP2R1 and CYP27A1 are key enzymes in the hydroxylation of vitamin D in the liver. CYP2R1 expression is significantly decreased in BA patients and was the leading cause of 25(OH)D deficiency rather than malabsorption, explaining the lack of response after oral supplementation of vitamin D [33]. The mechanism for this deficiency in BA patients is not precise. 

Lithocholic acid, a biliary compound that accumulates in cholestasis, can activate VDR and alter vitamin D signaling. Ligand-specific pleiotropy of VDR may explain the potentially opposing effects using a common pathway stimulated by different ligands (vitamin D and bile acid) [60].

The correlation between 25(OH)D deficiency and liver fibrosis in BA patients was confirmed in clinical studies [33]. Zhuang et al. [40] reported that the low serum level of 25(OH)D was correlated with the stage of fibrosis and serum level of PIIINP in BA patients. There were no associations between serum 25(OH)D level and liver function biomarkers, except for alkaline phosphatase (ALP). Ng et al. reported a negative correlation between 25(OH)D level and ALP, both pre-HPE and post-HPE. In a study including 33 children with BA after HPE, Peng et al. [61] reported a correlation between the low serum 25(OH)D level and severity of fibrosis evaluated by share wave elastography.

**Vitamin D and NAFLD**. NAFLD is a spectrum of progressive diseases that range from steatosis, inflammation, and fibrosis to cirrhosis and is now considered the most common chronic liver disease. Multiple factors are included in the pathogenesis of NALFD: metabolic syndrome, environmental risk factors, inherited susceptibility, and the risk factors are difficult to be assessed with precision. Due to the connections with metabolic syndrome, this condition was renamed Metabolic-Associated Fatty Liver Disease (MAFLD) [62].

Vitamin D regulates adipose tissue inflammation, liver fibrosis, aberrant fat accumulation in the liver, and insulin resistance [55,63,64,65,66]. Deficiency in vitamin D was associated with insulin-resistance-related diseases such as type 2 DM (T2DM), metabolic syndrome, and MAFLD [67].

Wang et al. [63] observed that NAFLD was more prevalent among the subjects with low levels of 25(OH)D, increasing the risk for NALFD in a specific population, explained by the genetic predisposition (a variant in the *VDBP* gene). Many studies [68,69,70] and meta-analyses [71,72,73] report that vitamin D deficiency is common in adults with NAFLD. 25(OH)D level is inversely correlated with aspartate aminotransferase (AST) and aspartate aminotransferase (ALT) [74]. A low level of 25(OH)D was associated with the severity of fibrosis in patients with NAFLD [75]. Other authors reported no significant association between a low level of 25(OH)D and the risk for NALFD in adults [76,77,78,79]. In children and adolescents, data are sparse. A recent meta-analysis, including eight articles (five cross-sectional and three case-control studies), supported the association of a low 25(OH)D level with NAFLD [80].

There is proof that vitamin D hydroxylation capacity is not perturbed in NALFD and does not represent the cause of vitamin D deficiency. Liver expression of CYP2R1 and CYP27A1 is preserved, and the expression of specific genes involved in vitamin D metabolism is not variable in NAFLD patients [36,68,81].

Studies on animal models report that vitamin D deficiency exacerbates the severity of NALFD histology [82,83]. There is a correlation between a low level of vitamin D with insulin resistance and liver inflammation by the upregulation of proinflammatory genes [82]. Vitamin D promotes adiponectin secretion in cultured adipocytes, and adiponectin has anti-inflammatory and insulin-sensitizing properties [84] and, together with VDR, is involved in the homeostasis of the other organs linked to MAFLD (gut and adipose tissue) [62]. Vitamin D3 and D2 protect against fatty liver, liver inflammation, and oxidative stress by inhibiting the p53-p21 signaling pathway and associated cell senescence, promoting the nuclear translocation of nuclear factor erythroid 2-related factor (NFE2L2), decreasing toll-like receptors (TLRs), repressing sirtuin, and activating the hepatocyte nuclear factor 4 alfa (HNF4 alfa) [67,82,85,86,87,88]. The role of vitamin D as an antifibrotic agent by inhibiting HSCs and expression of different profibrotic mediators was presented before. Moreover, the liver expression of VDR may modulate lipid accumulation by controlling the level of angiopoietin-like protein 3 and lipoprotein-lipase [89]. 

Despite all these experimental research results, interventional clinical trials found no beneficial impact of vitamin D supplementation in NAFLD patients regarding the biochemical markers, insulin resistance, adiponectin profile, liver histology, or ultrasonographic markers of liver injury [79,90,91,92,93,94,95].

**Vitamin D in chronic viral hepatitis**. Despite the decrease in the incidence of hepatitis B virus (HBV) infections due to the successful vaccination campaigns during the last decades, chronic HBV infection represents a public health burden and a significant cause of cirrhosis in adults.

Vitamin D deficiency is common in HBV-infected patients, and a low 25(OH)D level correlates with higher HBV replication [96,97]. Some studies contradict these findings and specify that the different phases of HBV infection may have different vitamin D impacts (related to host immunity) [98,99].

Regarding the role of vitamin D genetics in the outcome of HBV infection, studies report favorable outcomes with interferon (IFN) treatment in patients with a distinct genotype of CYP27B1 and VDR polymorphism [100]. Other studies support such correlations in specific populations of patients with HBV infection [101,102,103].

Chronic hepatitis C virus (HCV) infection represents a public health problem due to the high number of people infected and the increased risk for liver cirrhosis and hepatocellular carcinoma (HCC). The use of the new antivirals made the HCV infection treatment very efficient. Still, during PEGylate (Peg)-IFN treatment era, research was conducted to identify the risk factors for treatment failure. Vitamin D deficiency is common in patients with HCV infection, and data suggest that vitamin D could affect the treatment with Peg-IFN and ribavirin in those patients. In many countries, this is still the treatment approved for children under 12 years old.

Severe vitamin D deficiency was present in 30% of patients with HCV infection and cirrhosis, compared to 14% of non-cirrhotic HCV patients and 28% of patients with non-HCV cirrhosis [35]. Also, Miroliaee et al. [104] reports a higher prevalence of 25(OH)D deficiency in cirrhotic vs. non-cirrhotic patients (76.5% vs. 17.9%) and a correlation of low levels with more severe cirrhosis (Child-Pugh class B and C) in chronic hepatitis B, C, and AIH, findings that were supported by other studies [105,106]. A low 25(OH)D level was associated with the severity of fibrosis in HCV patients [107] and with an inadequate response to IFN-based therapy [56]. Vitamin D deficiency was correlated with a sustained virological response (SVR) in HCV genotype 2 and 3 patients [108]. Also, the same study reported that CYP27B1-1260 promotor polymorphism rs10877012 impacts 1,25(OH)2D level and SVR in HCV patients. Vitamin D may suppress HCV replication through oxidative stress pathways [35]. Gutierrez et al. [109] proved that vitamin D3, D2, and 1,25(OH)2D3 synergistically reduce HCV replication with IFN-alfa in cell culture. Also, vitamins D3 and D2 activate gene expression of CCL20 (macrophage inflammatory protein-3 alpha) and can aid in HCV clearance in vivo. There was also a strong upregulation of SCL30A10 in vitamin D-treated cells with the facilitation of zinc transport into cells. Zinc has a role in the innate and adaptative immune system, and it was proved that zinc inhibits HCV replication [110]. 

The clinical studies evaluating the 25(OH)D level and prediction of SVR response are controversial. One meta-analysis [111] revealed no association between 25(OH)D baseline level and SVR to Peg-IFN and ribavirin combination. Still, another meta-analysis, based on only three papers, supports the idea that low vitamin D level is linked to lower SVR [112]. There are also other studies without any correlation between the vitamin D level and treatment outcome [113,114,115]. Even though there is no proof that the baseline vitamin D level would influence the SVR in treatment with Peg-IFN and ribavirin, some studies reported that supplementation with vitamin D was beneficial [116,117,118,119].

**Vitamin D in autoimmune hepatitis (AIH).** Many studies have shown the importance of vitamin D in AIH, as these patients present a high prevalence of vitamin D deficiency [120,121]. It is well known that patients with low levels of 25(OH)D present severe interface hepatitis and advanced liver fibrosis or are more often non-responders to glucocorticoid therapy [122,123].

In AIH, both the genetic and non-genetic factors of the autoimmune process are related to vitamin D. Susceptibility to type 1 AIH is associated with B1*0301 and DRB1*0401 genes in Caucasian American or northern European people and with DRB1*0405 in Asian countries. Type 2 AIH is often associated with DRB1*07 alleles [124]. Vitamin D has an important effect on MHC class II antigen suppression in human mononuclear phagocytes and prevents proliferation and fibrosis processes through the effects on cytochrome p450, by increasing intracellular calcium flow, or by the effects on DNA polymerase α activity and cytoplasmic and nuclear protein kinase activity [124]. Vitamin also D regulates T-cell-mediated immunity. It decreased the cytotoxic T lymphocyte antigen-4 (CTLA-4) expression in monocytes and is a trigger for type 1 AIH [125]. In AIH, the number of monocytes increases, expressing an elevated level of regulated intracellular toll-like receptors (TLRs). Vitamin D decreases their activation, especially TLR-2, TLR-4, and TLR-9. Another role of vitamin D is in detoxification processes by increasing the expression of P450 cytochromes (CYP3A4, CYP2B6, and CYP2C9) [124]. In type 2 AIH with LKM antibodies, the activity of CYP2D6 is inhibited, affecting the oxidation; peroxidation; and/or reduction of vitamins, steroids, and xenobiotics metabolization [126]. Different VDR variants are also involved in AIH pathogenesis, primarily through the connection with fatty acid synthase (FAS) promoter variants, with some pro-inflammatory cytokines or liver fibrosis promoters [127,128,129]. In active AIH, the number of CD4+CD25high T cells is reduced and expresses low levels of forkhead helix transcription factor 3 (Foxp3). This transcriptional regulator plays an essential role in maintaining self-tolerance and preventing autoimmune diseases by regulating the function of CD4+ T regulatory cells (Tregs), a distinct lymphocyte with an inhibitory effect on the activation of the immune system [130]. The consequence of this Foxp3 reduction is decreased protective capacity against abnormal cell proliferations [124,130]. Tregs cells are more significant in AIH patients compared with control and correlate with the liver’s inflammatory activity [124,130].

Regarding the non-genomic role of vitamin D in AIH, it participates in the up-regulation of phosphatase 1 mitogen-activated protein kinase (MAPK) signaling pathways, thus regulating cytokine production. It is an important inhibitor of Gamma delta (γδ) T cells, a small subset of T cells with pro-inflammatory activity [124,131]. Vitamin D provides protection against the oxidative injuries caused by nitrite production, reduces the extent of lipid peroxidation, and stimulates the hepatic antioxidant system [124,131]. 

**Vitamin D in acute liver injury**. Supplementation with vitamin D may improve the necro-inflammation and apoptosis induced by hepatic ischemic-reperfusion injury in rats. This favorable response is attributed to attenuated TLR4 signaling, a potent activator of Kupffer cells. The findings of this study support the possible use of vitamin D supplementation before hepatic surgery as a simple and cost-efficient method for improving the outcome [36,132,133].

**Vitamin D in liver transplanted patients**. Most patients had a low 25(OH)D level before LT, and after transplantation, vitamin D deficiency was rare [134]. Some studies support that a low level of 25(OH)D before the LT may be linked with rejection episodes [135].

## 4. VDBP and Liver Disorders 

VDBP is synthesized by hepatic parenchymal cells, under the influence of estrogen, glucocorticoids, and inflammatory cytokines, but not by vitamin D. In all conditions involving tissue necrosis or injury (acute liver failure, ALF, septic shock, tissue traumatism), serum level of VDBP is significantly reduced. It is unknown at this time if VDBP is pathogenetically involved in the diseases or if it is just a consequence [25,28].

**VDBP in acute liver failure (ALF)**. Most studies describe a decrease of VDBP in patients with acute or CLD and its normalization after LT. However, the level of VDBP drops rapidly in ALF, as the liver synthesizes it, but also due to a rapid clearance of this protein in acute lesions. The decrease is not very specific, as it is found in many other acute liver injuries (vascular rupture, hemorrhage, viral and toxic hepatitis) or a variety of other acute illnesses, including cardiovascular, autoimmune, infectious diseases, organ failures or burns, and is considered a marker of poor prognostic [26,27,28,29,136]. In ALF, decreased level of VDBP will affect the actin-cleaning system, leading to a worse prognosis. Several studies confirm the involvement and usefulness as a marker in predicting potentially ALF fatal cases, with a low serum level of <100 mg/L of VDBP being inversely correlated with survival. Also, the serum level of VDBP correlates significantly with other parameters already used as prognostic markers (coagulation factors V, VII, INR or albumin) or with specific scores used to determine the need for transplantation in adults or children (King’s College, PELD/MELD score) [21,25,28,29,30,31,32,136,137]. The level of VDBP in ALF patients also varied with age and etiology. In a recent study performed on children, serum VDBP was significantly lower in neonates (0–28 days) and infants (1–12 months) compared with older children (1–14 years) and teenagers (14–18 years). In neonates and infants, ALF was caused by inborn errors of metabolism and infections, while autoimmunity and toxic hepatitis predominated at older ages. This difference correlates with the severity of hepatic necrosis, which is much more extensive in diseases in younger children. In adults, the level of VDBP also correlates with the etiology of ALF. Many studies report values significantly higher in patients with acetaminophen-induced ALF than in other causes of ALF [21,28,29,30,31,32,136,137].

**VDBP in CLD**. The level of VDBP is less decreased or is normal in CLD than in ALF, without consequences for vitamin D transport or bone metabolism [25,137]. This decreased level can be associated with other protein abnormalities, such as reduced caeruloplasmin or transferrin, regardless of etiology. The level of VDBP can be normal or even increased, especially in hemochromatosis or chronic active hepatitis. In patients with pre-existing liver injuries, a worsening of the damages is described with a new injuria caused by an increase of the pro-coagulating status because of an extra release of actin. Data about vitamin D serum levels and its usefulness as a marker in CLD are limited, with few studies reporting so far [25,32,137,138,139].

**VDBP in chronic viral hepatitis**. Most studies have been performed mainly in adults with chronic HBV infection, where vitamin D and VDBP have been shown to have implications for the evolution of the disease. Both low 25(OH)D and VDBP levels promote viral replication and fibrosis progression [139]. In children with chronic HBV infection, the association between vitamin D level, VDBP, HBV replication, or hepatic fibrosis was described in a large study by Huang. Serum levels of VDBP and 25(OH)D were decreased in children with HBV infection and progressive liver disease compared with healthy children. Also, in children with chronic HBV infection during the immune clearance phase (HBeAg positive, high serum of HBV-DNA, and increased transaminases) or those infected with genotype C, vitamin D and VDBP were decreased compared to children with inactive carrier phase (HBeAg positive, low serum HBV-DNA, and normal transaminases) or those infected with genotype B. This decrease in VDBP is most likely secondary to lower production in damaged hepatic cells, greater consumption of scavenging actin, and the viral replication that stimulates monocyte and neutrophil chemotaxis [139,140,141]. 

Regarding liver fibrosis and cirrhosis, the level of VDBP is correlated with the degree of hepatic fibrosis. Children with chronic HBV infection and stage 1 or 2 of fibrosis had higher VDBP levels, while the level decreased in patients with stage 3 fibrosis. Also, in adults with compensated liver cirrhosis after HBV infection, the serum level of VDBP was significantly lower than in healthy controls, and a direct correlation between plasma VDBP and fibrosis degree and Child-Pugh score has been described [136,137]. The exact mechanism of this correlation is not yet known, and it is only assumed that in the early stages of fibrosis, increased VDBP production is associated with the process of hepatocyte regeneration [136,137]. In another study, Thanapirom et al. evaluated the role of VDBP genetic polymorphism in response to treatment with Peg-IFN in patients with HBeAg-negative chronic HBV infection. Eight genotypes of vitamin D cascade genes, including CYP27B1 (rs10877012), DHCR7 (rs12785878), CYP2R1 (rs2060793, rs12794714), and GC (rs4588, rs7041, rs222020, rs2282679) were analyzed. The GC rs222020 TT genotype has been identified in patients with HBsAg clearance and ALT normalization after treatment with Peg-IFN [142].

In chronic HCV infection, even though vitamin D deficiency is rare, VDBP polymorphisms (especially rs7041 and rs4588) are frequently associated with lower levels of 25(OH)D and rapid fibrosis progression [143]. VDBP possesses many functions implicated in the modulation of the inflammatory response; therefore, its polymorphism determines the variability of the response to IFN therapy. It is involved in nonspecific immune response, protects against disseminated intravascular coagulation, transports lipids such as arachidonic acid and endotoxin, and stimulates the chemotactic activity of the C5a. This variability could be significant in identifying patients without response to antiviral therapy [143].

**VDBP in autoimmune liver diseases**. Genetic predisposition and environmental factors, including vitamin D metabolism, are the main factors responsible for triggering the immune process in autoimmune liver disease. In AIH, PBC or primary sclerosing cholangitis, vitamin D deficiency or VDR from the dendritic cells, and macrophages, seem to be among the important factors in triggering the immune process, the role of VDBP being less studied. In a recent study analyzing the VDBP level in children with ALF of various etiologies, we found that the level of VDBP in AIH was within the reference limits [25,28].

**VDBP and NAFLD**. It is well known that vitamin D deficiency predisposes to NAFLD or metabolic syndrome, but there are few data on the VDBP role in NAFLD. Specific variants of VDBP (mutation of VDBP-rs7041-G) have been shown to decrease the risk of NAFLD [63].

**VDBP in cirrhosis**. In patients with cirrhosis and liver synthesis deficits, the total VDBP starts to fall slightly. Interestingly, the reduction does not correlate with the disease’s severity and acuity. This is because the process of necrosis is not so intense; systemic inflammatory response syndrome is reduced compared to acute injuries and possibly due to increased levels of estrogen, which occurs in cirrhotic patients [25].

**VDBP in liver transplantation**. In patients with LT, the level of VDBP was initially low, associated with the cirrhosis degree, and then returned to normal levels after the transplant. During the first days after LT, the level of VDBP may decrease (in patients with normal values before transplantation) due to cold ischemia or surgical stress [144]. It seems that the level starts to balance after day 3 and reaches the normal value after more than 10 days. Regarding the immunosuppression used after LT, it does not influence the level of VDBP; on the contrary, corticosteroids increase the level of this protein. Some authors also described a high risk of acute cellular rejection and CMV infection in LT patients with a specific VDBP genotype [144].

## 5. Vitamin D Supplementation and Chronic Liver Diseases

The data reported until now suggest that vitamin D supplementation may be a beneficial potential therapeutic option in chronic liver diseases. Even though the association between vitamin D deficiency and the presence of MAFLD was proved, the clinical trials investigating vitamin D supplementation in these patients revealed controversial findings [67]. Some data suggest that the efficacy of vitamin D supplementation in MAFLD is linked to its role in glucose tolerance and insulin resistance. The best effect was young patients, with mild liver disease, without established diabetes and only in association with antifibrotic agents [67,145].

Also, one reason for the difficulty in proving the efficacy of vitamin D supplementation through nutrition in real life could be that exogenous sources are dependent on the individual dietary habits, use of vitamin D food fortification or different vitamin D supplements, but also due to other reasons linked to sun exposure. The advice for vitamin D supplementation in vitamin D deficiency situations presented in nutritional guidelines is meant for the general population [38]. For the patients with CLD, with a particular recommendation for those with cholestasis, cirrhosis, and steatosis, EASL suggests evaluating the 25(OH)D level and supplementing with oral vitamin D for those with levels under 20 ng/mL until reaching a serum level above the 30 ng/mL [146]. In cirrhotic patients with bone disease, depending on the severity of osteopenia, EASL Guidelines recommend a balanced diet and the supplementation with calcium (1000–1500 mg/d) and 25(OH)D (400–800 IU/d or 260 µg every 2 weeks) associated in severe cases with biphosphonates or new agents [146].

One crucial issue that should be considered when using vitamin D-fortified food is represented by the strategies to provide stability, bioaccessibility, and functionality of vitamin D. As mentioned before, significant individual variability of the response to vitamin D supplementation was reported. Moreover, the bioaccessibility of vitamin D depends on the food matrix. An essential role is played by lipids, proteins, fibers, and antioxidants. Future studies should focus on finding the best source for vitamin D administration [147].

## 6. Conclusions and Future Perspectives

As intense research during the last decades revealed the possible role of vitamin D in liver diseases, a deeper understanding of the vitamin D, VDR, and VDBP involvement in liver inflammation and fibrogenesis could represent the basis for the development of new strategies for diagnosis, prognosis, and treatment. Further studies with a larger population, in children and adults, with specific inclusion criteria, are needed to prove the benefit of vitamin D supplementation in patients with liver diseases.

## Data Availability

Not applicable.
